# Ocean Acidification-Mediated Food Chain Transfer of Polonium between Primary Producers and Consumers

**DOI:** 10.3390/toxics11010014

**Published:** 2022-12-23

**Authors:** Montaha Behbehani, Saif Uddin, Sam Dupont, Scott W. Fowler, Aysun U. Gorgun, Yousef Al-Enezi, Lamya Al-Musallam, Vanitha V. Kumar, Mohammad Faizuddin

**Affiliations:** 1 Environment Pollution and Climate Program, Kuwait Institute for Scientific Research, Safat 13109, Kuwait; 2 Department for Biological and Environmental Sciences, University of Gothenburg, Kristineberg 566, 451 78 Fiskebäckskil, Sweden; 3 Radioecology Laboratory, International Atomic Energy Agency (IAEA), 4 Quai Antoine 1er, 98000 Monaco, Monaco; 4 School of Marine and Atmospheric Sciences, Stony Brook University, Stony Brook, New York, NY 11794-5000, USA; 5 Institute of Nuclear Sciences, Ege University, 35100 Bornova/İzmir, Turkey; 6 Gulf Geoinformation Solutions, Hamariya Free Zone, P.O. Box 32223 Sharjah, United Arab Emirates

**Keywords:** phytoplankton, zooplankton, ocean acidification, bioaccumulation, fecal pellets

## Abstract

Phytoplankton and zooplankton are key marine components that play an important role in metal distribution through a food web transfer. An increased phytoplankton concentration as a result of ocean acidification and warming are well-established, along with the fact that phytoplankton biomagnify ^210^Po by 3–4 orders of magnitude compared to the seawater concentration. This experimental study is carried out to better understand the transfer of polonium between primary producers and consumers. The experimental produced data highlight the complex interaction between the polonium concentration in zooplankton food, i.e. phytoplankton, its excretion via defecated fecal pellets, and its bioaccumulation at ambient seawater pH and a lower pH of 7.7, typical of ocean acidification scenarios in the open ocean. The mass of copepods recovered was 11% less: 7.7 pH compared to 8.2. The effects of copepod species (n = 3), microalgae species (n = 3), pH (n = 2), and time (n = 4) on the polonium activity in the fecal pellets (expressed as % of the total activity introduced through feeding) was tested using an ANOVA 4. With the exception of time (model: F_20, 215_ = 176.84, *p* < 0.001; time: F_3_ = 1.76, *p* = 0.16), all tested parameters had an impact on the polonium activity (copepod species: F_2_ = 169.15, *p* < 0.0001; algae species: F_2_ = 10.21, *p* < 0.0001; pH: F_1_ = 9.85, *p* = 0.002) with complex interactions (copepod x algae: F_2_ = 19.48, *p* < 0.0001; copepod x pH: F_2_ = 10.54, *p* < 0.0001; algae x pH: F_2_ = 4.87, *p* = 0.009). The experimental data underpin the hypothesis that metal bioavailability and bioaccumulation will be enhanced in secondary consumers such as crustacean zooplankton due to ocean acidification.

## 1. Introduction

There has been a growing interest in understanding the effect of ocean acidification (OA) in modulating the metal uptake among the phytoplankton and zooplankton in the coastal and marine ecosystems [1,2,3,4,5,6]. Marine phytoplankton are primary producers in the ocean, forming the base of the marine food web. The oceanic sequestration of carbon dioxide is leading to a lowering of pH. The average oceanic pH has dropped to ~8.1, reaching the historical low for the past 2 million years [7]. With the increasing levels of atmospheric CO_2,_ oceanic pH is expected to drop by 0.3–0.4 units by the end of this century [8]. OA affects marine organisms in multiple ways, such as changes in acid-base balance, energy metabolism, redox balance, as well as the behavior of marine organisms [9,10,11]. For example, the growth of marine phytoplankton is affected by the increase in CO_2_ concentration in seawater [2,6,12,13,14,15,16]. 

One of the overarching effects of OA on phytoplankton communities is that phytoplankton will likely alter the chemical speciation of trace metals [4,17,18,19,20]. Phytoplankton concentrate metals and transuranic elements from surrounding waters [21,22,23,24,25]. Some of these metals such as iron (Fe), copper (Cu), and zinc (Zn) are essential for the growth and development of phytoplankton. In contrast, others such as cadmium (Cd), mercury (Hg), lead (Pb), and polonium (Po) have no known biological functions but are highly toxic at elevated concentrations [26,27]. Considering increased oceanic productivity under climate change [28,29], and the reported polonium concentration factors (CF) of 10^3^–10^4^ in microalgae from the Gulf [24,30], and even a higher CF of 10^5^ among Gulf copepods [31], it is quite probable to observe a trophic transfer of polonium to higher trophic levels in marine food chains [30].

Zooplankton are an important link between primary producers and fish. Copepods are primary consumers in the ocean and the most numerous metazoans, supporting the marine food web and also acting as a biological pump of carbon [32]. Any effect on the abundance and diversity of copepods due to ocean acidification is likely to have serious implications on the proper function of marine ecosystems. Studies have shown the lethality of increasing *p*CO_2_ levels on both surface and deeper copepod species [33]; however, the deeper copepods demonstrate greater tolerance to higher *p*CO_2_. It can be hypothesized that this higher tolerance is probably an adaptation to the high pCO_2_ environment where they live. It has been reported that ocean acidification and warming causes oxidative stress and a reduction in the reproductive capacity of *Acartia bifilosa* [34]; however, the magnitude of these responses varies between species and also varies spatially in different regions [33,35,36]. The Gulf ecosystem is somewhat unique for its temperature, salinity, nutrient-loading, and pH and has shown resilience to these changes (i.e. corals are surviving at a higher temperature in turbid water; a breeding ground for various fin and shellfish, are in areas reported to be exposed to potentially stressing conditions) [15,24,37,38]. 

This study was designed to understand the polonium transfer among the primary producers and primary consumers under lower pH conditions that might prevail due to increased oceanic CO_2_ sequestration considering the scenario of a 0.4 unit pH drop that is expected by the end of the century. The interest in understanding the behavior of polonium in a marine environment has been the focus of studies for the past five decades, since it results in an enhanced radiation dose to marine biota [39]. Studies have suggested that an anthropogenic contribution can enhance the ^210^Po concentrations considerably in the marine environment [40,41,42,43,44]. Marine organisms are capable of concentrating radionuclides in their tissues [39,43,45,46,47,48,49,50,51,52,53,54,55,56,57,58,59,60,61,62,63]; however, polonium elimination from zooplankton via their excretion of fecal pellets has been reported by Beasley et al. [64]. The concentration of polonium is of enormous interest because of its large contribution to the natural radiation dose and its high radiotoxicity [53,55]. 

In our previous experiment [4], we looked at polonium uptake in five microalgal species, i.e., *Thalassiosira weissflogi*, *Tetrasemis suecica*, *Isochrysis galbana*, *Chaetoceros muelleri*, and *Dunaliella salina* that are commonly used as a food source in the mariculture/aquaculture industry. 

This experimental study is designed to understand, under future oceanic pH as per IPCC RCP8.5, likely changes in ^210^Po concentration and possible uptake kinetics among the primary producers and consumers that are very important links in the marine food web. The microalgae with a known concentration of ^209^Po were used for feeding copepods to understand the assimilation rates, transfer, and turnover of this radionuclide in micro- crustacean zooplankton. 

## 2. Material and Methods

### 2.1. Sampling and Monoculture

The copepod samples were collected using a 50 µ plankton net with 0.6 m diameter and 2 m length. The samples were collected by vertical tows from 10–30 m depth to the surface in October 2019. All the samples obtained were placed in filtered seawater and immediately transported to the laboratory for sample identification and segregation to create a monoculture of *Acartia pacifica*, *Euterpina acutifrons*, and *Parvocalanus crassirostis*. The sample was emptied into a Bogorov counting chamber and examined under a stereomicroscope. Fine needles and pipettes were used to isolate the identified species for preparing a monoculture. Only adults were used for the experiment. Samples collected on a single day cruise from different areas were segregated at the lowest taxonomic level by specialists in copepod identification and following the characteristics of the species in an identification guide [65]. All the samples of single species from different areas were combined into a single beaker with filtered seawater. Once the monoculture was segregated, they were transferred into 1 L cylindrical enclosures in 50 L aquariums. 

### 2.2. Carbonate Chemistry

The pH on the total scale, alkalinity (A_T_ in µmol/kg), and salinity (in ‰) were measured at 12h, 24h, 36h, and 48h after the beginning of the experiment in each replicate. These data were used to calculate *p*CO_2_ (in µatm) using CO2SYS with constants from Mehrbach et al. [66] refitted by Dickson and Millero [67] and a temperature of 25°C. An ANOVA 2 statistical test was used to test the effect of target pH (8.2 and 7.7) and replicates (n = 3 per treatment) on the carbonate chemistry.

### 2.3. Experimental Exposure 

The experiment was carried out in 50 L aquaria. Copepods were housed in a 1 L cylindrical enclosure with a mesh of 45 µm at the cod end and a sealed bottom, enabling easy segregation of excreted fecal pellets. The stocking density of the experiment was 1000 individuals per enclosure. The experiment was conducted in triplicate for each of the *Parvocalanus crassirostis*, *Euterpina acutifrons*, and *Acartia pacifica* monocultures. The copepods were maintained at 25 ± 0.5 °C on a 16:8 h light–dark cycle and the water was gently aerated. All beakers were maintained at the required pH for 48 h to assess the Po transfer in copepods.

*Isochrysis galbana*, *Chaetoceros muelleri*, and *Dunaliella salina* labeled with ^209^Po were used as radiolabeled food to assess the transfer. The average ^209^Po concentration in each phytoplankton species was determined (Table 1). 

**Figure 1:**
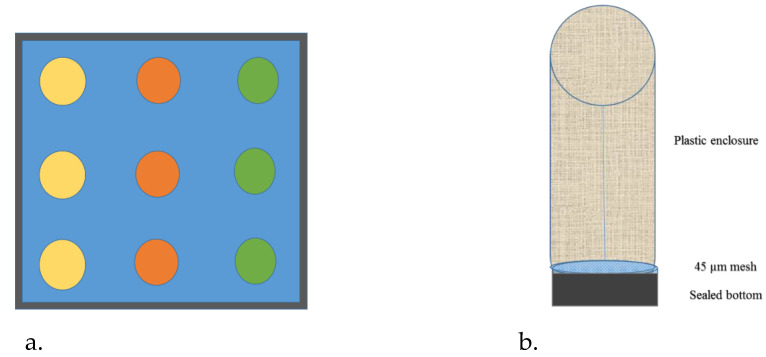
(**a**). Experimental setup of 3 cylindrical enclosures in a sealed compartment and 3 divisions in each aquaria. The color represents the type of copepod in triplicate. (**b**). The design of the enclosure with plastic wall and 45 µm base and sealed bottom.

Three replicates for each copepod and each algae (Figure 1) were taken considering 8.2 pH as a control as it represents the present-day ambient pH of the Gulf and Mediterranean waters, and 7.7 pH mimicking the worst case scenario for 2100 in these waters. The pH was regulated using the IKS Aquastar system by bubbling CO_2_ into the aquaria. 

The experimental setup consists of six aquaria with nine enclosures each, and each aquarium has a single pH condition and an algae type for the three copepod types. The microalgal food was added slowly with 10 mL of food being added twice a day to each cylindrical enclosure. The water turns green but as the microalgae were consumed, it again becomes clear. It was observed that the maximum quantity of fecal pellets was obtained within 60 min of feeding. The experiment was terminated after 48 h and all the copepods were filtered on a 0.45 µm filter paper and dried. The water was also analyzed.

Each sample of fecal pellets, phytoplankton, copepod, and water was digested using concentrated nitric acid, to allow determining the concentration of ^208^Po and ^209^Po. The samples were digested for at least 24 h, with occasional hydrogen peroxide additions to help in oxidizing the organic compounds. When a clear solution was obtained, it was allowed to evaporate to near dryness to remove HNO_3_. This concentrated residue was then dissolved in 100 mL of 0.5M HCl, and the solution placed on a magnetic stirrer at 30 °C for 24 h, following the procedure of Benoit and Hemond [68]. After the iron reduction with ascorbic acid, polonium (^209^Po and ^208^Po) in the solution were then spontaneously plated onto the surface of a 0.64-mm-thick silver disc (1.2 cm diameter) to be used as a thin alpha source for alpha spectrometry [69].

A Canberra forty-eight chamber alpha spectrometry system with passive ion-implanted silicon detectors (active area of 450 mm^2^, a background count of 2.3 per day, and a minimum depletion thickness of 90 µm) was used for ^208^Po and ^209^Po determination using the 5.110 MeV energy of ^208^Po and 4.877 MeV energy of ^209^Po alpha particle emission. The counting time was 24 h. Upon plating, the solution was converted to 9M HCl and passed through an ion-exchange column (DOWEX 1 × 8 100–200 mesh) to ensure the complete removal of the polonium tracers. The samples were stored for six months to allow the in-growth of sufficient ^210^Po from ^210^Pb for performing a second ^210^Po determination, however that is not reported here. 

As part of the analytical quality assurance procedures, reagent blanks, and an IAEA certified reference material were also analyzed with each batch of samples. Several aliquots of the IAEA 446–Baltic Sea Seaweed certified reference material (CRM) were analyzed for ^210^Po, and the concentration of ^210^Pb (through the determination of ^210^Po in secular radioactive equilibrium with ^210^Pb) was found to vary between 10.1 and 10.7 Bq kg^−1^ with a median value of 10.5 Bq kg^−1^. This result compared well with the ^210^Pb (^210^Po) information value of 10.9 Bq kg^−1^ and a 95% confidence interval of 10.2 to 12.0 Bq kg^−1^ provided in the CRM certificate. 

Seawater samples from the aquaria were analyzed after the exposure experiment using an established method [70]. The seawater sample was filtered using a 0.2 μm pore-size membrane filter and placed in a 1 L glass beaker. Ten mL of concentrated HCl was added to the sample to prevent radionuclides from adhering to the beaker wall. A spike of 100 µL of ^208^Po tracer was added, and the sample stirred for 3 h to ensure radiotracer mixing. Five mL of each 0.2 M KMnO_4_ and 0.3 M MnCl_2_ were added, and the solution was adjusted to pH 9 with 25% NH_4_OH. This solution was stirred for 3 h, and then the precipitate was allowed to settle out for a day. Most of the supernatant was carefully decanted so as to not disturb the precipitate, and the remainder was centrifuged. The centrifuged precipitate was recovered with 10 mL of 1% H_2_O_2_ in 5 M HCl from the centrifuge tube, taken to dryness on a hot plate, and the residue dissolved with 10 mL of 1% H_2_O_2_ in 2 M HCl. Polonium in this solution was then plated on a silver disc. This silver disc was used as a thin alpha source for alpha spectrometry and determination of ^209^Po in the aquaria sample. 

Mean ^209^Po concentrations in each microalgal species are given with the standard deviation of the sample (mean ± SD). Statistical analyses were performed using SAS. Differences between categories (copepod and algae species, pH, time, and replicate) were tested using ANOVA models followed by Scheffe’s post-hoc tests when relevant. All data are presented as mean ± standard error of mean (SEM). We have used SEM as it indicates how different the population mean is from the sample mean, and how much the sample mean would vary if the study is repeated using a new sample within a single population. The pH was kept constant using IKS Aquastar computers as well as the addition of bicarbonates when required. 

## 3. Results

### 3.1. Carbonate Chemistry

A summary of the parameters of the carbonate chemistry over the course of the experiment is summarized in Table 2. Target pHs were reached (pH 8.20 ± 0.01 and 7.69 ± 0.01) and significant effects between pH treatments were observed for pH, alkalinity, and *p*CO_2_. No significant differences were observed between replicates (Table 2). 

The ^209^Po recoveries were calculated by using ^208^Po tracer. The measured activities at 8.2 and 7.7 pH in fecal pellets, organisms, seawater fractions, and the activity initially added are presented in Table 3 and Table 4. The activity in fecal pellets, copepods, and water was subtracted from the total activity of algal food added to each experiment. The unaccounted activity is listed as loss, which might be due to adhesion on sides of the aquaria, losses in transfer, and possibly evaporation during the source preparation.

### 3.2. Copepods

**Figure 2:**
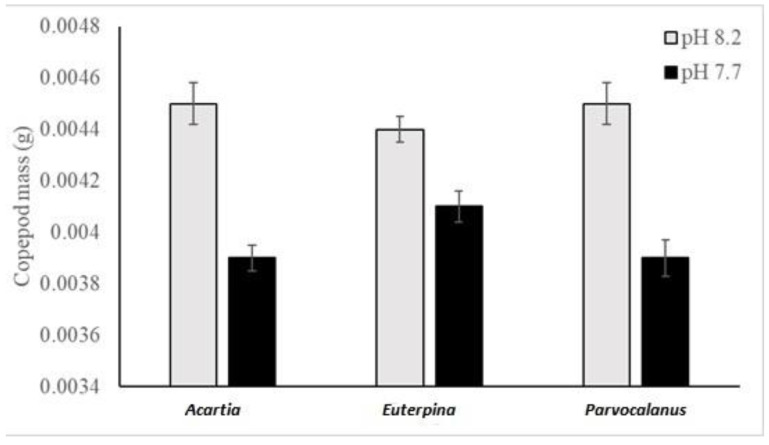
Copepod mass (in g) for the 3 copepod species (*Acartia pacifica*, *Euterpina acutifrons* and *Parvocalanus crassirostis*) and the two pHs (8.2 and 7.7). Results are presented as mean ± SEM.

The species had no significant effect on the mass of the organism (copepods) recovered after the experiment (ANOVA 2, model: F_5,53_ = 18.16, *p* < 0.0001; species: F_2_ = 0.38, *p* = 0.68). However, their mass was 11% lower at pH 7.7 as compared to pH 8.2 (F_1_ = 85.4, *p* > 0.0001) with no significant interaction between the two tested parameters (F_2_ = 2.40, *p* = 0.10; Figure 2). 

**Figure 3:**
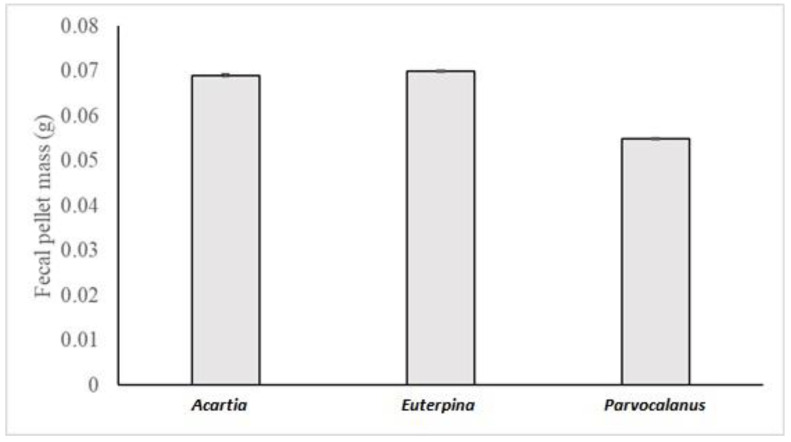
Fecal pellet mass (in g) for the 3 copepod species (*Acartia pacifica*, *Euterpina acutifrons* and *Parvocalanus crassirostis*). Results are presented as mean ± SEM.

The statistical analysis revealed that the weight of the fecal pellet was only significantly different between copepod species (ANOVA 4, model: F_20,215_ = 164.37, *p* < 0.0001; species: F_2_ = 1611.63, *p* < 0.0001) but not the microalgae species (F_2_ = 1.25, *p* = 0.29), pH (F_1_ = 1.55, *p* = 0.22), or time (F_3_ = 2.19, *p* = 0.09). A Scheffe’s post-hoc test revealed that the fecal pellet weight was significantly different between the three species. The difference in dry weight was small between *Acartia* and *Euterpina*, however it was 25% in *Parvocalanus* as compared to the two other species (Figure 3). 

### 3.3. Polonium

Effects of copepod species (n = 3), algae species (n = 3), pH (n = 2), and time (n = 4) on the polonium activity (expressed in % of the total activity introduced through feeding) in the fecal pellets were tested using an ANOVA 4. With the exception of time (model: F_20,215_ = 176.84, *p* < 0.001; time: F_3_ = 1.76, *p* = 0.16), all tested parameters had an impact on the polonium activity (copepod species: F_2_ = 169.15, *p* < 0.0001; algae species: F_2_ = 10.21, *p* < 0.0001; pH: F_1_ = 9.85, *p* = 0.002) with complex interactions (copepod x algae: F_2_ = 19.48, *p* < 0.0001; copepod x pH: F_2_ = 10.54, *p* < 0.0001; algae x pH: F_2_ = 4.87, *p* = 0.009). 

**Figure 4:**
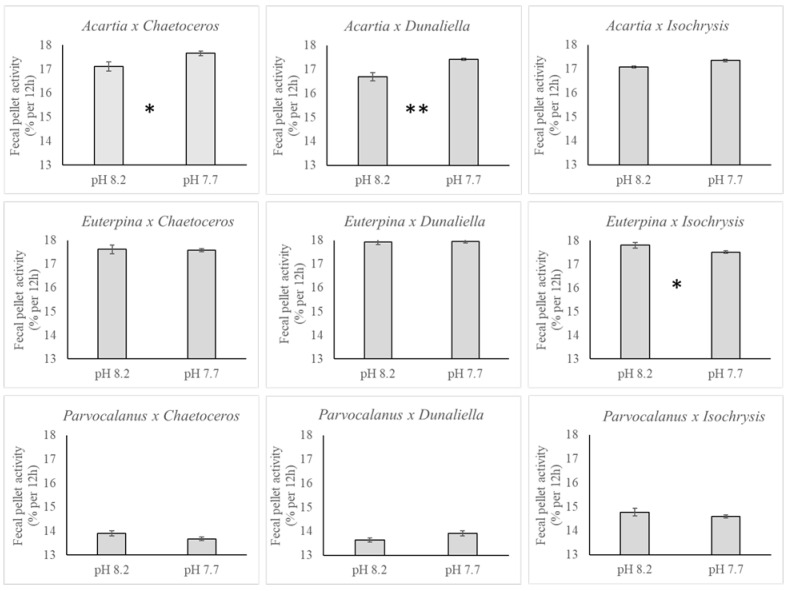
Effect of pH on polonium activity in the copepod fecal pellets (in % per 12 h) for each combination of copepod (*Acartia pacifica*, *Euterpina acutifrons* and *Parvocalanus crassirostis*) and microalgae species (*Chaetoceros muelleri*, *Dunaliella salina and Isochrysis galbana*). Results are presented as mean ± SEM. Significant difference between pH treatments are indicated with * (ANOVA; *p* < 0.05) and ** (*p* < 0.01).

The average polonium activity in the fecal pellets collected from each of the three copepods show higher concentrations at lower pH of 7.7 with the exception of *Parvocalanus* on *Isochrysis* feed. The effect of pH for each combination of copepod and algal species is presented in Figure 4. A significant 3 to 4% increase in polonium activity is observed under pH 7.7 as compared to pH 8.2 across the experiment. 

**Figure 5:**
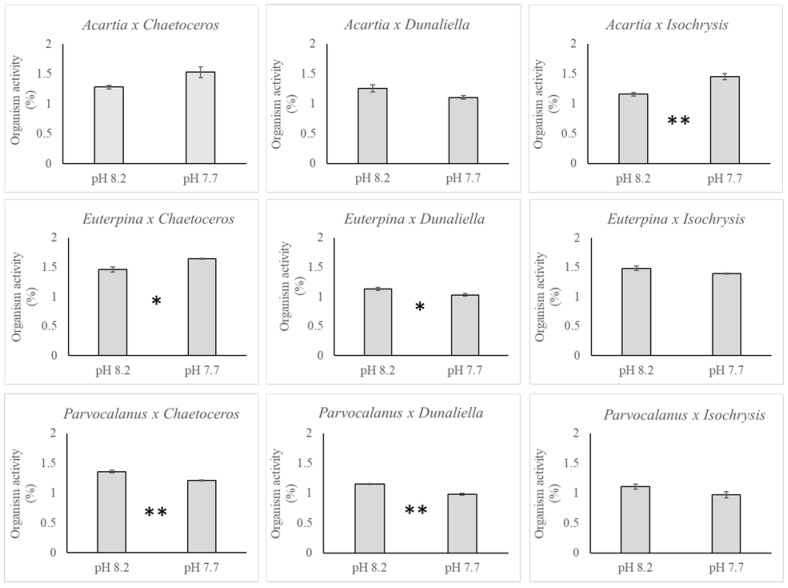
Effect of pH on polonium activity (in %) in the copepod for each combination of copepod species (*Acartia pacifica*, *Euterpina acutifrons*, and *Parvocalanus crassirostis*) and microalgae species (*Chaetoceros muelleri*, *Dunaliella salina*, *and Isochrysis galbana*). Results are presented as mean ± SEM. Significant difference between pH treatments are indicated with * (ANOVA; *p* < 0.05) and ** (*p* < 0.01).

The relative activity of polonium in the copepods at the end of the experiment ranged between 0.97 and 1.64% of the total activity provided through food. This activity was significantly different between copepod species (ANOVA 3; model: F_17,53_ = 27.48, *p* < 0.0001; copepod: F_2_ = 56.14, *p* < 0.0001), algae species (F_2_ = 99.62, *p* < 0.0001), pH (F_1_ = 86.74, *p* < 0.0001), and the interaction between copepod and algae species (F_4_ = 14.44, *p* < 0.0001). The general trend was a higher polonium concentration at lower pH of 7.7 with the exception of *Acartia* and *Euterpina* on *Isochrysis*. The effect of pH for each combination of copepod and algae species is presented in Figure 5.

## 4. Discussion

**Figure 6:**
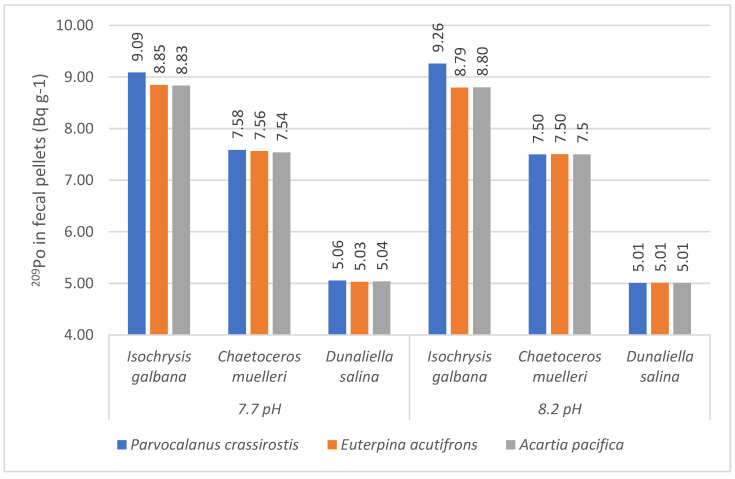
Average ^209^Po concentration in fecal pellets at 7.7 and 8.2 pH in three copepod species for the three algal feeds.

The average ^209^Po concentration in fecal pellets of *Parvocalanus crassirostis* were 9.26 and 9.09 Bq g^−1^, 7.50 and 7.58 Bq g^−1^, and 5.01 and 5.06 for *Isochrysis galbana*, *Chaetoceros muelleri*, and *Dunaliella salina* feeding at 8.2 and 7.5 pH (Figure 6). The average concentrations for *Isochrysis galbana* were skewed because of a single outlier, on the other three occasions the concentration were 8.80 Bq g^−1^. The ^209^Po excretion with fecal pellets was 1–2% higher at lower pH in *Parvocalanus crassirostis*. 

The average ^209^Po concentration in fecal pellets of *Euterpina acutifrons*, at 8.2 and 7.7 pH were 8.79 and 8.85 Bq g^−1^, 7.50 and 7.56 Bq g^−1^, and 5.01 and 5.03 Bq g^−1^, for *Isochrysis galbana*, *Chaetoceros muelleri*, and *Dunaliella salina* (Figure 6). The values were lower at 7.7 pH but the difference in ^209^Po concentrations were between 0.5–0.8% among the algal food sources.

Among *Acartia pacifica* fecal pellets the ^209^Po concentration varied between 8.80 and 8.83 Bq g^−1^, 7.50 and 7.54 Bq g^−1^, and 5.01 and 5.04 Bq g^−1^ for *Isochrysis galbana*, *Chaetoceros muelleri*, and *Dunaliella salina* feeds at 8.2 and 7.5 pH, respectively. The ^209^Po fecal pellet concentration at 7.7 exceeds 8.2 by ~0.5% among different feed types.

The average ^209^Po concentration in fecal pellets was 0.5–2% higher at 7.7 pH compared to 8.2 pH with the single exception of *Parvocalanus crassirostis* ingesting *Isochrysis galbana* (Figure 6). The experimental data generated in this study supports the hypothesis that metal bioavailability is likely to change under an ocean acidification scenario. We draw a parallel with Fowler [71] who reported surface-bound Pb is unassimilated in zooplankton and is therefore eliminated in fecal pellets. We are speculating that this observation might also be the case with polonium in our experiment, which led to higher ^209^Po in fecal pellets in the experiment and could be due to the fact that much of the ^209^Po was bound to the surface of the ingested phytoplankton and might not have gone in the cytoplasm of the phytoplankton.

**Figure 7:**
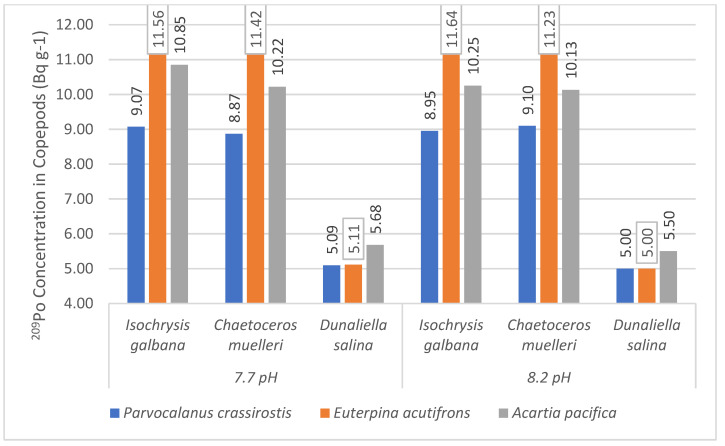
Average ^209^Po bioaccumulation in *Parvacanus crassitoris*, *Euterpina acutifrons*, and *Acartia pacifica* ingesting different phytoplankton species at 7.7 and 8.2 pH.

The ^209^Po bioaccumulation was higher in *Parvocalanus crassirostis*, *Euterpina acutifrons*, and *Acartia pacifica* at 7.7 pH compared to 8.2, with only the two exceptions of *Acartia pacifica* and *Euterpina acutifrons* feeding on *Isochrysis galbana* (Figure 7). The polonium retention efficiency was 0.97–1.64%, suggesting a lower assimilation of this non- essential actinide. It is interesting to compare the ^210^Po in these copepods, the ^210^Po concentration in *Euterpina acutifrons*, *Acartia pacifica,* and *Parvocalanus crassirostis* were 0.17–0.18 Bq g^−1^ (dw), 0.26–0.27 Bq g^−1^ (dw)*,* and 0.61–0.65 Bq g^−1^ (dw), respectively, which is substantially lower than those observed in this experiment; however, the concentration factors for these organisms were 10^5^. Drawing inference from the fact that higher bioaccumulation was observed at lower pHs, we assume that CFs will be higher under acidifying conditions. 

The experimental data indicate that the ingested polonium was rapidly defecated by all the three copepods, and the sinking fecal pellets would depurate surface waters of polonium. In addition, the question remains, will theses fecal pellets become part of the food chain of detritivores. Unfortunately, in this experiment, we have not looked at the residence time and retention of polonium in the fecal pellets, which will be interesting to examine under ocean acidification conditions. There is a likelihood of polonium being retained in fecal pellets, which would result in relatively short residence times in surface waters. 

The high concentrations of metals in marine sediments and water are known for various marine areas worldwide [72,73,74,75,76]. With anthropogenic activities, including sewage and industrial discharges, dredging spoils, and natural sources as river discharges and atmospheric inputs, a temporal increase in metal concentrations is eminent in seawater and sediments. Studies have demonstrated the bioaccumulation and toxicity of ^210^Po in various marine organisms from phytoplankton, zooplankton, crustaceans, fish, etc., across the trophic food chain [21,23,24,25,30,31,49,50,51,52,53,54,57,58,64,77,78,79,80,81,82,83,84,85,86]. Studies have reported that copepods create feeding currents and selectively ingest food particles [87,88,89]. Kadiene et al. [20] demonstrated that metal uptake in copepods from the dissolved phase was significantly higher than metals from their diet. 

## 5. Conclusions

This experimental study has provided data to underpin possible ^210^Po transfer processes at the base of the marine food chain under ocean acidification conditions. Considering increased oceanic productivity under climate change, and significant bioaccumulation of polonium in microalgae from the Gulf, and even a higher concentration among Gulf copepods, it is quite probable to observe a polonium reduction in surface water due to the bioconcentration mechanism and abundance of the phytoplankton and zooplankton. On the contrary, the higher polonium concentration and significant quantities of fecal pellet production in mass is likely to result in polonium enrichment in the deeper levels and in bottom sediments due to the downward sinking of fecal pellets. The likelihood of deep water and bottom-sediment enrichment becoming part of the bottom-dwelling marine organism’s and detritivore’s food chains should be considered and could result in an enhanced dose to these benthic organisms. 

A 0.5–2% enrichment of ^209^Po at lower pHs might result in considerable vertical flux considering that with the amount of fecal pellets produced over the life time of a copepod will be at least 2-3 orders of magnitude greater than its mass. The assimilation fraction of ingested polonium was 0.97–1.64% and a slightly higher bioaccumulation was noted at a lower pH of 7.7 compared to 8.2; however, this study also supports the hypothesis of negligible retention of actinides, unlike the retention of other metals such as Zn (47%), Cd (30%), and Ag (17%) [80]. 

Assuming a similar biological behavior of ^209^Po and ^210^Po, it is quite interesting to note that significantly higher ^209^Po concentrations in the *Parvocalanus crassirostis*, *Euterpina acutifrons*, and *Acartia pacifica* under the experimental conditions compared to ^210^Po in same species in their natural environment, where the enrichment was 100,000 times. 

Not only was the assimilation rate higher at a lower pH, the excretion of polonium was also higher at 7.7 pH compared to 8.2 pH. Polonium elimination in fecal pellets of zooplankton is a well-established phenomenon and has been known for decades. The higher concentration of polonium in copepod fecal pellets at a lower pH is likely to result in the efficient depletion of polonium in surface waters and its downward vertical flux through the water column. The retention of polonium in fecal pellets was roughly comparable to the concentration in the copepods, but the production rate of fecal pellets is quite high. This study supports the hypothesis that primary producers are likely to provide an enhanced dose of polonium to primary consumers, such as crustacean zooplankton. It would be quite interesting to assess the concentration of polonium bound to surfaces compared to that integrated in cytoplasm in phytoplankton, as the two fractions are likely to result in different assimilation rates in copepods. 

## Figures and Tables

**Table 1:** Average ^209^Po concentration in water with microalgae.

Microalgae	^209^Po Concentration (Bq mL^−1^)
*Isochrysis galbana*	0.088 ± 0.004
*Chaetoceros muelleri*	0.075 ± 0.003
*Dunaliella salina*	0.050 ± 0.002

**Table 2:** Summary of the carbonate chemistry: measured pH on the total scale, alkalinity (A_T_ in µmol/kg), salinity (in ‰), and calculated *p*CO_2_ (in µatm). Difference between target the two target pHs and between replicates was tested using an ANOVA 2. Results are presented as mean ± SEM.

	pH 8.2	pH 7.7	Model		pH		Replicate	
			F_5,23_	*p*	F_1_	*p*	F_4_	*p*
pH	8.20 ± 0.01	7.69 ± 0.01	3828.0	<0.0001	19,137.3	<0.0001	1.1	0.41
A_T_	2890 ± 1	2610 ± 1	55,171.0	<0.0001	275,848.0	<0.0001	1.4	0.26
Salinity	42 ± 0	42 ± 1	1.0	0.45	.	.	.	.
*p*CO_2_	307.0 ± 1.4	1131.0 ± 9.9	1394.7	<0.0001	6969.6	<0.0001	1.17	0.36

**Table 3:** ^209^Po concentration in the experimental samples at 8.2 pH.

			*Isochrysis galbana*			*Chaetoceros muelleri*			*Dunaliella salina*		
	Sample	Time	Mass (g)	Activity (mBq)	Concentration (Bq/g)	Mass (g)	Activity (mBq)	Concentration (Bq/g)	Mass (g)	Activity (mBq)	Concentration (Bq/g)
*Parvocalanus crassirostis*	Fecal Pellet	12	0.0517	550	10.64	0.0533	399	7.49	0.0562	281	5.00
	Fecal Pellet	24	0.0566	498	8.80	0.0545	409	7.50	0.0533	267	5.01
	Fecal Pellet	36	0.0590	519	8.80	0.0561	421	7.50	0.0545	273	5.01
	Fecal Pellet	48	0.0583	513	8.80	0.0549	412	7.50	0.0537	269	5.01
	Copepod	48	0.0038	34.0	8.95	0.0040	36.4	9.10	0.0039	19.5	5.00
	Water-microalgae	48		1213			1176			808	
	Ʃ algal food added	48	40 mL	3520		40 mL	3000		40 mL	2000	
	Loss	48		193			146.6			82.5	
*Euterpina acutifrons*	Fecal Pellet	12	0.0721	634	8.79	0.0672	504	7.50	0.0727	364	5.02
	Fecal Pellet	24	0.0688	605	8.79	0.0695	521	7.50	0.0698	349	5.00
	Fecal Pellet	36	0.0730	642	8.79	0.0733	550	7.50	0.0709	355	5.01
	Fecal Pellet	48	0.0710	625	8.80	0.0718	539	7.51	0.0731	366	5.01
	Copepod	48	0.0042	48.9	11.64	0.0039	43.8	11.23	0.0041	20.5	5.00
	Water-microalgae	48		807			733			439	
	Ʃ algal food added	48	40 mL	3520		40 mL	3000		40 mL	2000	
	Loss	48		158.1			109.2			106.5	
*Acartia pacifica*	Fecal Pellet	12	0.0670	590	8.81	0.0711	533	7.50	0.0683	342	5.01
	Fecal Pellet	24	0.0698	614	8.80	0.0736	552	7.50	0.0685	343	5.01
	Fecal Pellet	36	0.0663	583	8.79	0.0644	483	7.50	0.0631	316	5.01
	Fecal Pellet	48	0.0702	618	8.80	0.0685	514	7.50	0.0674	337	5.00
	Copepod	48	0.0040	41	10.25	0.0038	38.5	10.13	0.0040	22	5.50
	Water-microalgae	48		910			779			551	
	Ʃ algal food added	48	40 mL	3520		40 mL	3000		40 mL	2000	
	Loss	48		164			100.5			89.0	

**Table 4:** ^209^Po concentration in the experimental samples at 7.7 pH.

			*Isochrysis galbana*			*Chaetoceros muelleri*			*Dunaliella salina*		
	Sample	Time	Mass (g)	Activity (mBq)	Concentration (Bq/g)	Mass (g)	Activity (mBq)	Concentration (Bq/g)	Mass (g)	Activity (mBq)	Concentration (Bq/g)
*Parvocalanus crassirostis*	Fecal Pellet	12	0.0572	524	9.16	0.0512	390	7.62	0.0567	290	5.11
	Fecal Pellet	24	0.0545	513	9.41	0.0548	417	7.61	0.0542	273	5.04
	Fecal Pellet	36	0.0572	509	8.90	0.0581	438	7.54	0.0551	278	5.05
	Fecal Pellet	48	0.0575	510	8.87	0.0554	419	7.56	0.0542	272	5.02
	Copepod	48	0.0043	39	9.07	0.0046	40.8	8.87	0.0045	22.9	5.09
	Water-microalgae	48		1268			1147.2			769.1	
	Ʃ algal food added	48	40 mL	3520		40 mL	3000		40 mL	2000	
	Loss	48		157			148			95	
*Euterpina acutifrons*	Fecal Pellet	12	0.0689	612	8.88	0.0682	518	7.60	0.0722	361	5.00
	Fecal Pellet	24	0.0693	610	8.80	0.0698	524	7.51	0.0703	354	5.04
	Fecal Pellet	36	0.0707	623	8.81	0.0699	531	7.60	0.0709	358	5.05
	Fecal Pellet	48	0.0698	621	8.90	0.0711	536	7.54	0.0725	364	5.02
	Copepod	48	0.0045	52	11.56	0.0043	49.1	11.42	0.0044	22.5	5.11
	Water	48		869			729.9			440.8	
	Ʃ algal food added	48	40 mL	3520		40 mL	3000		40 mL	2000	
	Loss	48		133			112			99.7	
*Acartia pacifica*	Fecal Pellet	12	0.0688	610	8.87	0.0703	529	7.52	0.0691	348	5.04
	Fecal Pellet	24	0.0693	612	8.83	0.0721	546	7.57	0.0695	349	5.02
	Fecal Pellet	36	0.0686	605	8.82	0.0695	523	7.53	0.0688	346	5.03
	Fecal Pellet	48	0.0699	616	8.81	0.0692	521	7.53	0.0694	351	5.06
	Copepod	48	0.0047	51	10.85	0.0045	46	10.22	0.0044	25	5.68
	Water	48		868			737			507	
	Ʃ algal food added	48	40 mL	3520		40 mL	3000		40 mL	2000	
	Loss	48		158			98			74	

## Data Availability

The statements, opinions and data contained in all publications are solely those of the individual author(s) and contributor(s) and not of MDPI and/or the editor(s). MDPI and/or the editor(s) disclaim responsibility for any injury to people or property resulting from any ideas, methods, instructions or products referred to in the content.
